# Genome-wide identification of *GH3* genes in *Brassica oleracea* and identification of a promoter region for anther-specific expression of a *GH3* gene

**DOI:** 10.1186/s12864-020-07345-9

**Published:** 2021-01-06

**Authors:** Jiseong Jeong, Sunhee Park, Jeong Hui Im, Hankuil Yi

**Affiliations:** Department of Biological Sciences, College of Biological Science and Biotechnology, Chungnam National University, Daejeon, 34134 Republic of Korea

**Keywords:** *Brassica oleraceea var. oleracea*, TO1000, *Gretchen Hagen 3*, *GH3*, Anther, Promoter

## Abstract

**Background:**

The *Gretchen Hagen 3* (*GH3*) genes encode acyl acid amido synthetases, many of which have been shown to modulate the amount of active plant hormones or their precursors. *GH3* genes, especially Group III subgroup 6 *GH3* genes, and their expression patterns in economically important *B. oleracea* var. *oleracea* have not been systematically identified.

**Results:**

As a first step to understand regulation and molecular functions of Group III subgroup 6 *GH3* genes, 34 *GH3* genes including four subgroup 6 genes were identified in *B. oleracea* var. *oleracea*. Synteny found around subgroup 6 *GH3* genes in *B. oleracea* var. *oleracea* and *Arabidopsis thaliana* indicated that these genes are evolutionarily related. Although expression of four subgroup 6 *GH3* genes in *B. oleracea* var. *oleracea* is not induced by auxin, gibberellic acid, or jasmonic acid, the genes show different organ-dependent expression patterns. Among subgroup 6 *GH3* genes in *B. oleracea* var. *oleracea*, only *BoGH3.13–1* is expressed in anthers when microspores, polarized microspores, and bicellular pollens are present, similar to two out of four syntenic *A. thaliana* subgroup 6 *GH3* genes. Detailed analyses of promoter activities further showed that *BoGH3.13–1* is expressed in tapetal cells and pollens in anther, and also expressed in leaf primordia and floral abscission zones.

**Conclusions:**

Sixty-two base pairs (bp) region (− 340 ~ − 279 bp upstream from start codon) and about 450 bp region (− 1489 to − 1017 bp) in *BoGH3.13–1* promoter are important for expressions in anther and expressions in leaf primordia and floral abscission zones, respectively. The identified anther-specific promoter region can be used to develop male sterile transgenic *Brassica* plants.

**Supplementary Information:**

The online version contains supplementary material available at 10.1186/s12864-020-07345-9.

## Background

The *Gretchen Hagen 3* (*GH3*) gene was first identified in *Glycine max* (soybean) as an early response gene, which is transcriptionally induced in less than 30 min by treatment of auxin plant hormone [1]. Later studies have found that *GH3* genes are found in diverse plant species including mosses and fern, but not in two model algae, *Chlamydomonas reinhardtii* or *Volvox carteri* [2–7]. Like acyl CoA synthetases, non-ribosomal peptide synthetases, and luciferases in ANL superfamily proteins, GH3 proteins conjugate combinations of amino acids and acyl acids in two-step reactions [8, 9]. In the first half-reaction involving ATP and acyl acid, adenylated acyl acid is produced and pyrophosphate is released. In the second half-reaction, adenylated acyl acid intermediate reacts with amino acids, resulting in the release of acyl acid-amino acid amido conjugate and adenosine monophosphate. For example, *Arabidopsis thaliana* (Arabidopsis) GH3.11, jasmonate (JA) resistant 1 (JAR1), and Arabidopsis GH3.17, reversal of sav 2 (VAS2), catalyze the production of JA-isoleucine and indole acetic acid (IAA)–glutamate, respectively [10, 11].

GH3 proteins are involved in various developmental processes and environmental responses in plants, by modulating the activities or availabilities of plant hormones and related compounds, including precursors of plant hormones [12]. Abnormal expressions caused by null mutation or hyper- and mis-expression lead to various phenotypic defects. In Arabidopsis, *atgh3.11* (*jar1*) mutant does not produce bioactive JA-Isoleucine and defective in JA signaling, while *atgh3.17* (*vas2*) mutant over-accumulates free IAA at the expense of IAA-glutamate [11, 13]. In addition, *atgh3.12* (*avrPphB susceptible 3* (*pbs3*)) mutant was found to be more susceptible to bacterial pathogens because production of isochorismoyl glutamate, the precursor of salicylic acid (SA), catalyzed by PBS3, is compromised [14]. Over-expression of *AtGH3.6* (*Dwarf in Light 1* (*DFL1*)) or *AtGH3.2* (*Yadokari 1* (*YDK1*)), which are induced by auxin, causes hyper-sensitivity to light treatment leading to dwarfism [15, 16]. Over-expression of *AtGH3.5* (*WES1*), which is induced by treatment of abscisic acid and SA, as well as auxin, leads to auxin resistant phenotypes [17]. In various plants, important roles played by plant GH3 enzymes have also been demonstrated: nodule numbers and sizes in soybean [18], resistance to *Xanthomonas* bacteria in citrus [19], drought and salt tolerance in cotton [20], and fruit softening in kiwi [21], were shown to be affected by *GH3* gene expressions.

Phylogenetic analyses show that plant *GH3* genes can be clustered into 3 groups (GroupI~ III) based on overall amino acid sequences or 8 subgroups (subgroup 1 ~ 8) based on acyl acid-binding site sequences of Arabidopsis, rice, soybean, maize, *Selaginella*, and moss GH3 proteins [7, 10, 12, 22]. However, only Group I and II *GH3* genes have been identified in Gramineae genomes [23–25]. Using GH3 enzymes in various plant species, preferential substrates of GH3 enzymes in terms of acyl acids and amino acids have been determined [8, 14, 18, 22, 26–30]. In addition, a systematic evaluation of sixty GH3 enzymes from Arabidopsis, grape, rice, *Physcomitrella*, and *Selaginella* also revealed that not all the enzymes encoded by Group I *GH3* genes are involved in JA signaling and 12 out of 16 enzymes encoded by Group II *GH3* genes display clear substrate preferences for IAA among three acyl acid substrates - jasmonate, IAA, and 4 hydroxybenzoate (4-HBA) [31]. In case of Group III *GH3* enzymes, which are encoded by the largest *GH3* group in the plant genomes, no clear substrate preferences were established, except AtGH3.9 or OsGH3.13 for IAA and Arabidopsis PBS3 for 4-HBA. In case of Group III subgroup 6 GH3s, only AtGH3.15 in Arabidopsis was shown to have substrate preference for indole butyric acid (IBA), the auxin precursor [28]. Although decrease in IBA-mediated root elongation inhibition and lateral root formation were observed in transgenic plants constitutively expressing *AtGH3.15*, in vivo function(s) of other subgroup 6 *GH3* genes have yet to be determined. In rapeseed (*Brassica napus*) and its diploid ancestors, Chinese cabbage (*Brassica rapa*) and cabbage (*Brassica oleracea var. capitata*), up to sixty-six GH3-coding genes have been identified [32, 33]. However, detailed study of GH3-coding genes in kale-type *Brassica* species (*Brassica oleracea var. oleracea*), TO1000, which serves as an excellent model for important vegetable crops in *Brassica oleracea* with various morphological and phytochemical traits [34], have not been performed yet.

The anther is a part of the stamen, the male reproductive organ in plants, and is connected to the flower receptacle by a filament, which is the other part of the stamen [35, 36]. Anther development is divided into two phases, culminating in the release of pollen grains, the male gametophytes in plants. Microsporogenesis, the first phase, includes establishment of anther morphology, cell and tissue differentiation, and meiosis of microspore mother cells. Tetrads of haploid microspores produced by meiotic divisions of diploid pollen mother cells are released as distinct unicellular microspores into locules by a mixture of enzymes produced from tapetum cells, which also provide nutrients and pollen wall materials for developing pollens [37, 38]. During microgametogenesis, the second phase, differentiation of microspores into pollen grains and tissue degeneration occur for the release of pollens. Microgametogenesis starts with the expansion of the microspore, which is often found with the formation of one large vacuole [39]. This involves movement of the microspore nucleus from the center of the cell to a position close to the cell wall, where the microspore produces two unequal cells, a large vegetative cell and a small generative cell, in a process called pollen mitosis (PM) I. Then, the generative cell, which is spatially separated from the pollen grain wall and engulfed by the vegetative cell, undergoes another round of cell division, called PM II [37]. Depending on whether PM II happens before or after pollen dispersal from the anther, the pollens are called tricellular or bicellular pollen [40]. Plant hormones - JA, auxin, gibberellic acid (GA), and ethylene – are known to play important roles in stamen maturation, locule opening, anther dehiscence, and pollen viability during stamen and pollen development [35, 41–43].

To expand our knowledge on the regulation and molecular functions of Group III *GH3* genes in plants – especially those in subgroup 6 whose functions are still elusive – *GH3* genes in kale-type *B. oleracea* var. *oleracea* were identified genome-wide, and expression patterns of subgroup 6 *GH3* genes were investigated. It was found that subgroup 6 *GH3* genes in *B. oleracea* var. *oleracea*, composed of four genes showing synteny with closely related Arabidopsis subgroup 6 *GH3* genes, are not induced by auxin, GA, and JA treatment, but have different organ expression patterns. *BoGH3.13–1*, a subgroup 6 *GH3* gene, is specifically expressed in tapetal cells in anther and pollens when microspores, polarized microspores, and bicellular pollens are produced, as well as in leaf primordia and floral abscission zones. Promoter bash experiments revealed that a 62 base pairs (bp) DNA sequence, − 340 to − 279 bp upstream of *BoGH3.13–1* start codon, is required for anther-specific expression, while a ~ 450 bp region (− 1489 to − 1017) is necessary for expression in leaf primordia and floral abscission zones.

## Results

### Thirty-four *GH3*-encoding genes (*BoGH3*s) are present in *B. oleracea* var. *oleracea*

In the Ensembl Plants database (http://plants.ensembl.org/index.html), protein sequences of 55 *GH3* candidate genes in kale-type *B. oleracea* showed similarities to the 19 Arabidopsis GH3 proteins [10]. Among these, 34 GH3 proteins were found to have intact GH3 domains (pfam03321) and considered as GH3 proteins (Table S1; Figure S1). Although identical genomic sequences were used for annotation, only 30 *B. oleracea* GH3 candidate proteins, including two with truncations in GH3 domains, were found to have significant similarities to Arabidopsis GH3s in NCBI database (NCBI, http://ncbi.nlm.nih.gov) [34]. The 34 BoGH3 proteins with the intact GH3 domains in Ensembl Plants database include all 28 putative GH3 proteins with the intact GH3 domains identified in NCBI database (Table S1). For proteins showing different protein sequences between two databases, such as BoGH3.12–2 and BoGH3.17–1, NCBI protein models were adopted in our study because they are supported by RNA-seq data in NCBI. While 34 GH3 protein-coding genes were identified from *B. oleracea* var. *oleracea* in our study, 25 and 29 GH3 protein-coding genes were previously reported for cabbage-type *B. oleracea* var. *capitata* in the comparison with *B. napus* genes by two independent studies, respectively [32, 33].

**Fig. 1:**
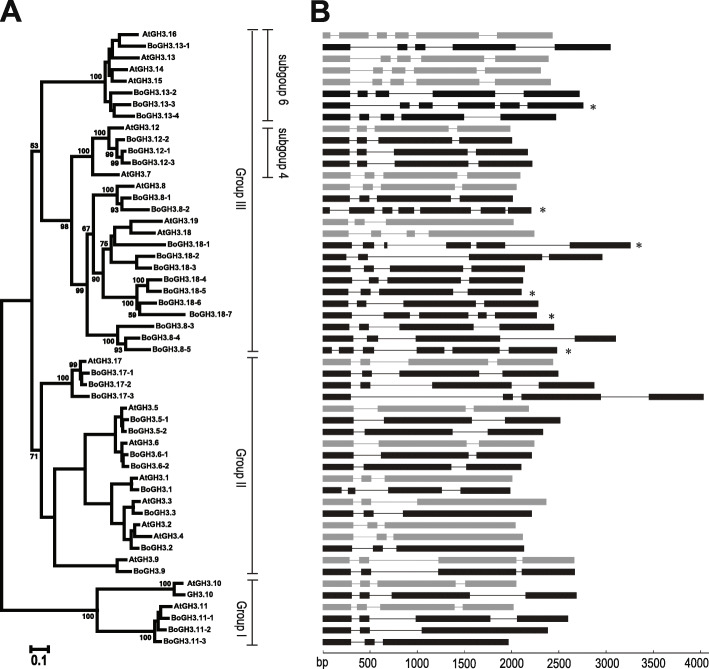
Phylogenetic relationships and exon/intron structures of GH3 proteins in Arabidopsis and *B. oleracea* var. *oleracea*. **a** Phylogenetic analysis of GH3 family members in Arabidopsis and *B. oleracea* var. *oleracea*. The percentage of trees in which the associated taxa clustered together is shown next to the branches. The phylogenetic tree is drawn to scale, with branch lengths measured in the number of substitutions per site. Bootstrap test percentages of 1000 replicates are shown next to the branches. **b** Gene structures for Arabidopsis and BoGH3 proteins were generated by gene structure display servers (http://gsds.cbi.pku.edu.cn/Gsds_about.php). Note that exons indicated here do not contain untranslated regions. Astertisks indicate exon/intron structures of genes, which were annotated only in Ensembl Plants. The black boxes and lines represent exons and introns in *B. oleracea* var. *oleracea GH3* genes, while the gray boxes and lines represent exons and introns in Arabidopsis *GH3* genes. Groups (I~ III) and subgroups (4 & 6) of GH3 proteins were designated based on the Staswick et al. (2002) and Westfall et al. (2012), respectively [10, 12]

Similar to previous phylogenetic analyses of GH3 proteins including cabbage-type *B. oleracea* var. *capitata*, phylogenetic clustering of Arabidopsis and BoGH3 proteins demonstrated that BoGH3 proteins can be divided into three groups (Group I, II, and III) (Fig. 1a) [6, 10, 32, 33]. It was found that Group I consists of two Arabidopsis and four BoGH3 proteins, while Group II consists of eight Arabidopsis and 11 BoGH3 proteins. In the case of Group III, nine Arabidopsis GH3s and 19 BoGH3 proteins were clustered together. In general, exon/intron structures of *BoGH3* genes were same to closely related counterparts in Arabidopsis with some exceptions (Fig. 1b). For example, four protein-coding exons were detected for *BoGH3.1* in Group II, based on the distribution of RNA-seq reads in NCBI database, while three protein-coding exons of *AtGH3.1* is reported in TAIR JBrowse (https://jbrowse.arabidopsis.org/). In case of *BoGH3.11–2* and *BoGH3.11–3*, which are closely related to *AtGH3.11* (*JAR1*) with four protein-coding exons, only three exons supported by RNA-seq reads were observed. Structural differences were also observed for five *BoGH3* genes (*BoGH3.8–2*, *BoGH3.8–5*, *BoGH3.13–3*, *BoGH3.18–1*, and *BoGH3.18–7*) that were identified only in Ensembl Plants.

### Synteny is observed for group III subgroup 6 *GH3* genes between Arabidopsis and *B. oleracea* var. *oleracea*

**Fig. 2:**
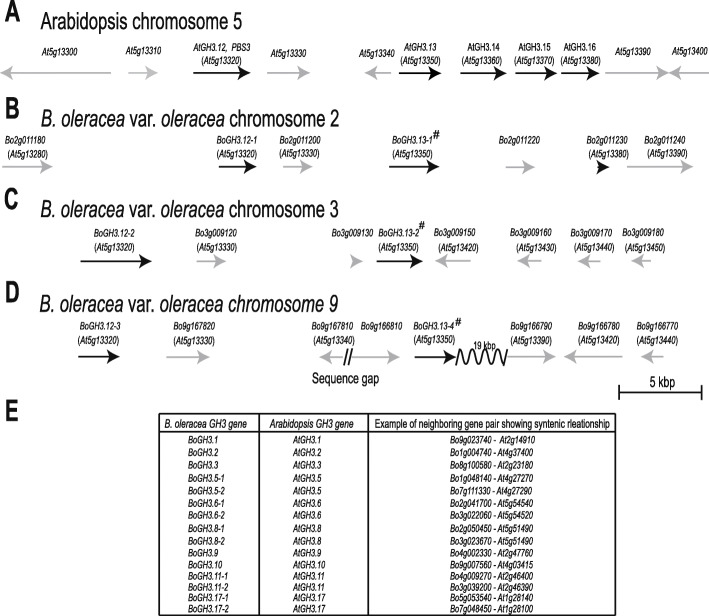
Syntenies are found between genomic regions around Arabidopsis *AtGH3.13* and corresponding regions in *B. oleracea* var. *oleracea*. Each panel shows gene organization, in which *GH3* and non-*GH3* genes from start to stop codons are indicated by black and gray arrows, respectively. Direction of each arrow shows that of gene transcription. **a** The gene organization on *Arabidopsis* chromosome 5 around *AtGH3.12*. **b-d** The gene organizations of *B. oleracea* var. *oleracea* chromosome 2 near *BoGH3.12–1,* chromosome 3 near *BoGH3.12–2*, and chromosome 9 near *BoGH3.12–4*. Arabidopsis genes showing sequence similarities to *BoGH3* genes are indicated in parenthesis below *B. oleracea* var. *oleracea* gene names. The *BoGH3* genes similar to Arabidopsis *AtGH3.13 ~ AtGH3.16* gene cluster are indicated with sharp (#) symbols. *Bo2g011230* in (B) encodes a truncated protein with a sequence similarity to Arabidopsis *GH3* genes in the cluster. **e** Syntenic relationships detected between other *BoGH3* genes in *B. oleracea* var. *oleracea* and Arabidopsis

In *B. oleracea* var. *oleracea*, 4 out of 34 Group III BoGH3 proteins (BoGH3.13–1, BoGH3.13–2, BoGH3.13–3, and BoGH3.13–4) show a close relationship with Arabidopsis subgroup 6 GH3 proteins (Fig. 1a). While the four *BoGH3* genes are found on different chromosomes, four Arabidopsis *GH3* genes (*AtGH3.13*, *AtGH3.14*, *AtGH3.15*, and *AtGH3.16*) in the same subgroup are located within 15,000 bp genomic region on Arabidopsis chromosome 5 (Fig. 2a). When genes located around Arabidopsis and *B. oleracea* var. *oleracea* subgroup 6 *GH3* genes were compared, syntenies were detected around the *AtGH3.13* ~ *AtGH3.16* cluster and three *BoGH3* genes (*BoGH3.13–1*, *BoGH3.13–2*, and *BoGH3.13–4*) (Fig. 2 b-d). In the upstream of three *BoGH3* genes, *Bo2g011200* (Fig. 2b), *Bo3g009120* (Fig. 2c), and *Bo9g167820* (Fig. 2d) showing sequence similarities to *At5g13330*, an *RAP2.6 L* transcription factor found upstream of the *AtGH3.13* ~ *AtGH3.16* cluster, were identified (Fig. 2a). Moreover, *BoGH3.12–1*, *BoGH3.12–2*, and *BoGH3.12–3*, which are clustered with *AtGH3.12 (PBS3*) in the phyologenetic tree as Group III subgroup 4 *GH3* genes, were also found further upstream, same to *AtGH3.12 (PBS3*) located upstream of the *AtGH3.13* ~ *AtGH3.16* cluster. Consistent with the syntenic relationships in these genomic regions, sequence similarities were also observed downstream of the Arabidopsis *GH3* cluster and the three *BoGH3* genes on different chromosomes (Fig. 2b–d): *Bo2g011240* and *Bo9g166790* show sequence similarity to *At5g13390*, *No Exine Formation 1*. In addition to six subgroup 4 and subgroup 6 *BoGH3* genes showing synteny (Fig. 2b – 2D), analyses for remaining 28 *BoGH3* genes revealed that 15 more *BoGH3* genes have syntenic relationships with *AtGH3* genes (Fig. 2e).

### Subgroup 6 *BoGH3* genes are not induced by auxin treatment in the seedling stage

**Fig. 3:**
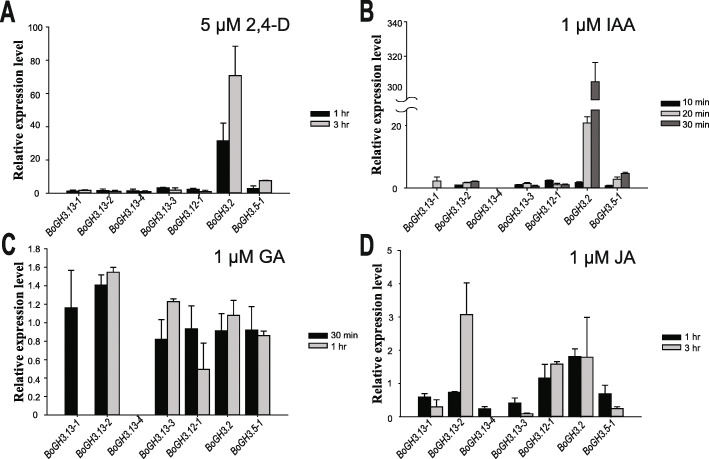
Subgroup 6 *BoGH3* genes are not induced by auxin at the seedling stage. Relative expression levels of four subgroup 6 *BoGH3* genes and three selected *GH3* genes in other subgroups in response to treatments of 5 μM 2,4-D (**a**), 1 μM IAA (**b**), 1 μM GA (**c**), and 1 μM JA (**d**) were determined by qRT-PCR experiment with *Actin* control. The expression level of mock condition was set to value 1 and used as reference to compare expression level changes after hormone treatments. Bar graphs show average relative expression values with standard errors (SE). Averages values of two independent results for 2,4-D or JA treatments are shown, while representative results are shown for IAA or GA treatments. Bar graphs for genes without any significant amplification are not included

In Arabidopsis, auxin treatment can induce transcription of some *GH3* genes, such as *AtGH3.2* (*YDK1*), *AtGH3.5* (*WES1*), and *AtGH3.6* (*DFL1*) [15–17]. However, expression conditions and functions of *GH3* genes in other plants are largely unknown. To gain insights on the expression patterns and functions of four *B. oleracea* var. *oleracea* subgroup 6 *GH3* identified in this study, we determined whether these genes can be induced by plant hormones and found that none of subgroup 6 *BoGH3* genes were significantly induced by auxin (synthetic 2,4-Dichlorophenoxy acetic acid (2,4-D) or natural IAA), GA or JA treatment at the seedling stage, except *BoGH3.13–2* that is weakly induced by JA (Fig. 3). One of subgroup 4 *BoGH3* gene, *BoGH3.12–1*, also did not show expression changes responding to hormone treatments. In contrast, transcriptional inductions by auxin were evident for *BoGH3* genes included as positive controls (*BoGH3.2* and *BoGH3.5–1*), which are closely related auxin-inducible Arabidopsis *GH3* genes [16, 17].

### *BoGH3.13–1* is strongly expressed in stamen at a specific stage during flower development

**Fig. 4:**
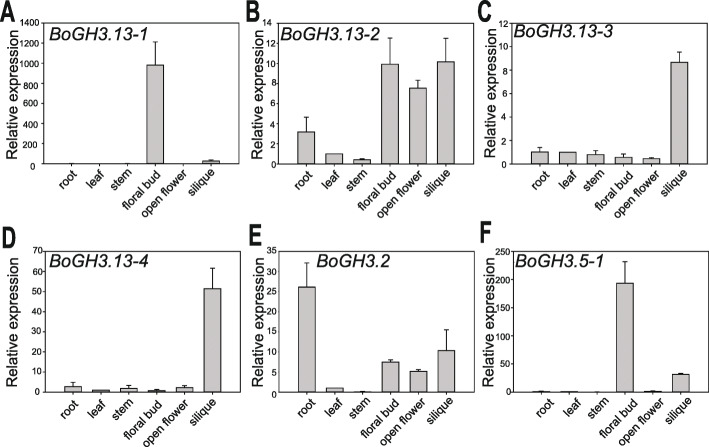
qRT-PCR results showing expression patterns of selected *BoGH3* genes, including four subgroup 6 *BoGH3* genes. Relative expression levels of *BoGH3.13–1* (**a**), *BoGH3.13–2* (**b**), *BoGH3.13–3* (**c**), *BoGH3.13–4* (**d**), *BoGH3.2* (**e**), and *BoGH3.5–1* (**f**) were determined by qRT-PCR experiment with *Actin* control in different organs and/or developmental stages. Bar graphs show average relative expression values with SEs. The expression level of leaf was set to value 1 and used as reference to compare expression levels in different organs

For four subgroup 6 and two auxin-inducible *GH3* genes in *B. oleracea* var. *oleracea*, relative expression patterns in six different organs - root, leaf, stem, floral bud, opened flower, and silique - were determined. Among four subgroup 6 *BoGH3* genes, *BoGH3.13–1* was found to be most strongly expressed in floral bud, although significant expression was also observed in silique compared to that in leaf (Fig. 4a). Only negligible expressions of *BoGH3.13–1* were detected in other organs, including open flowers. For the other three subgroup 6 *BoGH3* genes, the strongest expression was commonly found in siliques (Fig. 4 b-d), while comparable expressions in floral bud and open flower were also observed for *BoGH3.13–2* (Fig. 4b). For auxin-inducible *BoGH3.2* and *BoGH3.5–1*, which were included as comparison, distinct relative expression patterns were detected: *BoGH3.2* and *BoGH3.5–1* were found to be most strongly expressed in root and floral bud, respectively (Fig. 4 e & f). For three subgroup 4 *BoGH3* genes, stronger expressions were commonly observed in roots (Figure S2).

**Fig. 5:**
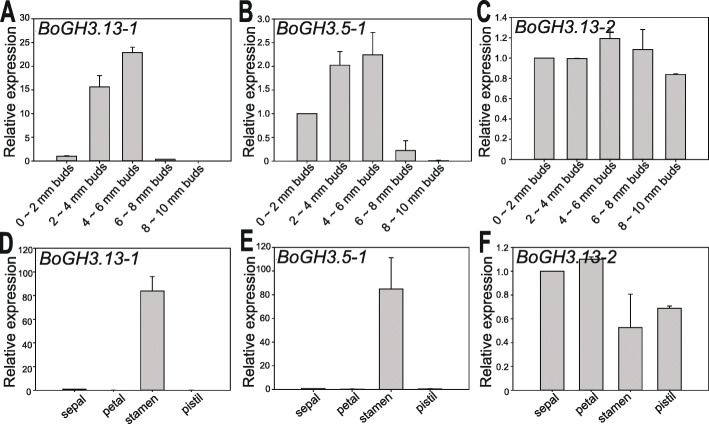
*BoGH3.13–1* and *BoGH3.5–1* are strongly expressed in anther. Steady-state expression levels of *BoGH3.13–1* (**a**), *BoGH3.5–1* (**b**), and *BoGH3.13–2* (**c**) in developing floral buds and those of *BoGH3.13–1* (**d**), *BoGH3.5–1* (**e**), and *BoGH3.13–2* (**f**) in sepal, petal, stamen, and pistil of 4 ~ 6 mm floral buds were determined with qRT-PCR. Bar graphs show average relative expression values with SEs. The expression level of 0 ~ 2 mm buds (**a-c**) and that of sepal (**d-e**), which were normalized to that of *ACTIN*, were set to value 1 and used as reference

For *BoGH3.13–1* and *BoGH3.5–1*, which show strong preferential expressions in floral bud (Fig. 4 a & f), it was also determined whether expressions of these genes are temporally regulated during floral bud development. When the expression levels were monitored for developing floral buds sorted by lengths (Figure S3), which reflect the progress of flower development [44], both genes showed stronger expression when bud lengths are about 2 to 6 mm, although *BoGH3.13–1* in subgroup 6 GH3 showed more dramatic expression changes by developmental progress than *BoGH3.5–1* (Fig. 5 a & b). In 4 ~ 6 mm-long floral buds, where the two genes are most strongly expressed, almost exclusive expression was detected in stamen among sepal, petal, stamen, and pistils (Fig. 5 d & e). In contrast, no significant developmental and organ-specific expression differences were observed for *BoGH3.13–2*, another subgroup 6 *BoGH3* that are constitutively expressed in floral buds, open flowers, and siliques (Figs. 4b, 5c & f).

### *BoGH3.13–1* and *BoGH3.5–1* are expressed in tapetum and pollen grains

**Fig. 6:**
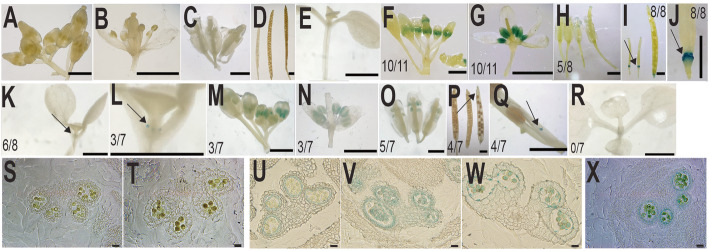
GUS staining patterns in Arabidopsis transgenic plants with two *BoGH3* promoter::*GUS* transgene. **a-e** Samples from wild-type Arabidopsis plants (WT) - floral buds (**a**), a dissected floral bud (**b**), open flowers (**c**), siliques (**d**), and 8-day old seedling (**e**). **f-l** Samples from *BoGH3.13–1 (− 1489 ~ − 1)::GUS* transgenic plants - floral buds (**f**), a dissected floral bud (**g**), open flowers (**h**), siliques (**i-j**), and 8-day old seedling (**k-l**). **m-r** Samples from *BoGH3.5–1 (− 1496 ~ − 1)::GUS* transgenic - floral buds (**m**), a dissected floral bud (**n**), open flowers (**o**), siliques (**p-q**), and 8-day old seedling (**r**). Transverse sections of GUS-stained floral buds of WT plants (**s-t**). Transverse sections of GUS-stained floral buds of *BoGH3.13–1 (− 1489 ~ − 1)::GUS* (**u-w**). Transverse sections of GUS-stained floral buds of *BoGH3.5–1 (− 1496 ~ − 1)::GUS* (**x**). Dividends and denominators of fractions in the pictures are transgenic plants with the GUS staining and all the transgenic plants examined, respectively. Arrows indicate GUS stained parts. Scale bars in (**a-r**): 1 mm. Scale bars in (**s-x**): 20 μm

To narrow down spatial expression patterns of stamen-expressed *BoGH3.13–1* and *BoGH3.5–1*, we generated transgenic plants, in which *GUS* (*β-glucuronidase*) reporter genes are expressed under the control of about 1500 bp putative promoter sequences of these *BoGH3* genes. *BoGH3.13–1 (− 1489 ~ − 1)::GUS* and *BoGH3.5–1 (− 1496 ~ − 1)::GUS* are two transgenic plants, in which *− 1489 ~ − 1* and *− 1496 ~ − 1* bp DNA sequences upstream of *BoGH3.13–1* and *BoGH3.5–1* start codon, respectively, are fused to *GUS* reporter genes. In *BoGH3.13–1 (− 1489 ~ − 1)::GUS*, *GUS* expression was observed in anthers of developing floral buds (Fig. 6 f & g), consistent with the qRT-PCR (quantitative reverse transcription polymerase chain reaction) results (Figs. 4 & 5). Weak GUS stainings in some stigmas were found to be caused by stigma-attached pollens (Fig. 6h). GUS staining was also observed in siliques, but only in the floral organ abscission regions of petals, sepals, and stamens (Fig. 6 i & j). In addition, GUS expression was detected in leaf primordia of *BoGH3.13–1 (− 1489 ~ − 1)::GUS* seedlings (Fig. 6 k & l). In *BoGH3.5–1 (− 1496 ~ − 1)::GUS*, GUS expression was detected in developing anthers and unfertilized ovule or aborted seeds (Fig. 6m-q), but not in seedling leaf primordia (Fig. 6r). To further define the spatial expression patterns of *BoGH3.13–1* and *BoGH3.5–1* in anther, cross-sectioned floral buds were examined and specific expression in tapetum cells and pollen grains were detected for both genes (Fig. 6u-x). In *BoGH3.13–1 (− 1489 ~ − 1)::GUS*, GUS staining seems to appear in the tapetum first and pollens later (Fig. 6u & v).

### *BoGH3.13–1* and *BoGH3.5–1* are most strongly expressed around when polarized microspores are generated

**Fig. 7:**
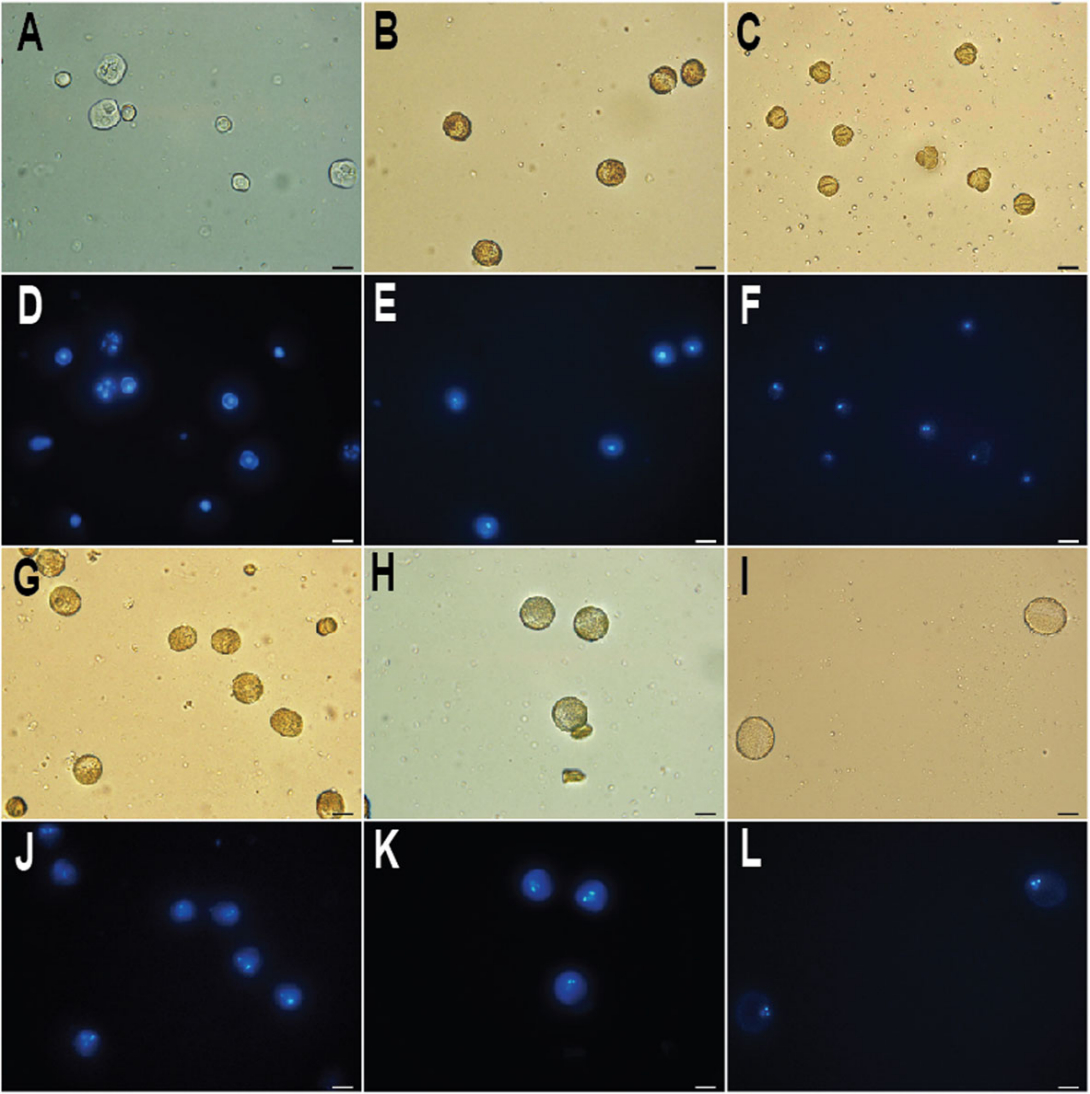
DAPI-stained developing pollen grains in *B. oleracea* var. *oleracea* floral buds. Bright-field images of pollens in less than 2 mm floral buds (**a**), 2 ~ 4 mm buds (**b**), 4 ~ 6 mm buds (**c**), 6 ~ 8 mm buds (**g**), 8 ~ 10 mm buds (**h**), and open flowers (**i**). Fluorescence images of DAPI-stained pollens in less than 2 mm floral buds (**d**), 2 ~ 4 mm buds (**e**), 4 ~ 6 mm buds (**f**), 6 ~ 8 mm buds (**j**), 8 ~ 10 mm buds (**k**), and open flowers (**l**). Scale bar: 20 μm

To investigate which milestone events in microsprogenesis or microgametogenesis occur in pollens when *BoGH3.13–1* and *BoGH3.5–1* are expressed (Fig. 5), developing pollens were collected from floral buds and open flowers. Based on the numbers and organization of 4′,6-diamidino-2-phenylindole (DAPI)-stained nuclei, it was found that tetrads and microspores are observed in less than 2 mm floral buds (Fig. 7 a & d), in which the two anther-expressed *GH3* genes, *BoGH3.13–1* and *BoGH3.5–1*, are weakly expressed (Fig. 5). In 2 ~ 6 mm floral buds, in which the two anther-expressed *GH3* genes are most strongly expressed, microspores, polarized microspores, and bicellular pollens were observed (Fig. 7b-c & e-f). While bicellular and tricellular pollens were observed in 6 ~ 8 mm buds, only tricellular pollens were observed in 8 ~ 10 mm buds and opened flowers (Fig. 7g- l). These data show that *BoGH3.13–1* and *BoGH3.5–1* are strongly induced when polarized microspores are mainly produced during early microgametogenesis [45, 46].

### One hundred eighty-six bp region upstream of *BoGH3.13–1* is sufficient for anther-specific expression

**Fig. 8:**
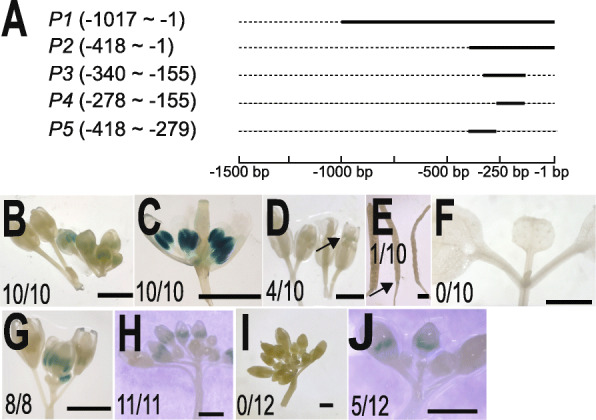
Representative GUS staining patterns to define a promoter region directing anther-specific expression of *BoGH3.13–1*. **a** Genomic DNA regions used in transgenic lines for promoter analysis. Names of transgenic plants used are written in italic and different regions upstream of *BoGH3.13–1* start codon to direct *GUS* reporter expression are indicated in parentheses. **b-f** GUS staining of floral buds (**b**), a dissected floral bud (**c**), open flowers (**d**), siliques (**e**), and 8-day old seedling (**f**) of *P1* transgenic plants, and floral buds from *P2* (**g**), *P3* (**h**), *P4* (**i**), and *P5* (**j**) are shown. Arrows indicate GUS-stained pollen (**d**) or floral abscission zones in a silique (**e**). Dividends and denominators of fractions in the pictures are numbers of transgenic plants with the GUS staining and all the transgenic plants examined, respectively. Scale bars: 1 mm

DNA sequences responsible for tissue-specific expression of *BoGH3.13–1* was investigated with different DNA regions upstream of the start codon (Fig. 8a). When P1, in which − 1017 ~ − 1 bp region was fused upstream of *GUS* reporter gene, was used to generate *P1* transgenic plants, *GUS* expressions in anthers and pollens were still detected (Fig. 8b-d), but those in floral abscission zones and leaf primordia were lost, except one case showing GUS staining in the floral abscission zone (Fig. 8 e & f). When P2 (− 418 ~ − 1) and P3 (− 340 ~ − 155), without − 155 ~ − 1 bp putative 5′ untranslated region based on RNA-seq reads in SRX209697 (NCBI), were used, anther-specific GUS expressions were found to be maintained (Fig. 8 g & h). While *P4* (− 278~ − 155) did not show GUS expression in all twelve lines, five out of twelve *P5* (− 418 ~ 279) showed GUS expression, suggesting sixty-two bp region (− 340 ~ − 279) in P3 sequence is important for anther-specific expression of *BoGH3.13–1* (Fig. 8 i & j).

## Discussion

Thirty-four GH3-coding genes of kale-type *B. oleracea* var. *oleracea*, which have intact GH3 domains, were identified from Ensembl plants database (Fig. 1a). Among these, 28 gene models were also found in NCBI database, which had used the identical genomic sequence for annotation [34]. The discrepancy in *BoGH3* gene numbers between Ensembl plants and NCBI database may result from the use of different gene prediction algorithms or validations. Recently, twenty-nine GH3 protein-coding gene models related to cabbage-type *B. oleracea* var. *capitata GH3* genes were identified from the investigation of genomic sequence of *B. napus* [32, 33], and twenty-eight genes were found to have intact GH3 domains and meet our criteria, while *Bol042635* was found to encode a truncated GH3 domain with only 224 amino acids (Table S2) [33]. Among 34 BoGH3 coding-genes reported in this study, putative orthologs of cabbage-type *B. oleracea* were identified for 19 genes, but clear orthologous relationship could not be determined for the other 15 *B. oleracea* var. *oleracea GH3* genes, based on amino acid sequence identities of over 95%. Considering 6 *B. oleracea* var. *oleracea GH3* genes, whose expression could not be confirmed in NCBI database, are included in these 15 cabbage-type *GH3* genes without putative orthologs, we speculate these 6 genes are pseudogenes and lost in *B. oleracea* var. *capitata*. In addition, orthologs of 9 cabbage-type *B. oleracea GH3* genes could not be determined in *B. oleracea* var. *oleracea* (Table S2).

Group III subgroup 6 *GH3* genes in *B. oleracea* var. *oleracea* and Arabidopsis seem to have evolved by duplications. Four Arabidopsis *GH3* genes in subgroup 6 are located within 15 kbp region on the same Arabidopsis chromosome, while 4 *BoGH3* genes in the same subgroup are located on 4 different chromosomes of *B. oleracea* var. *oleracea* (Fig. 2). Syntenies found between genomic regions around the subgroup 6 *AtGH3* and *BoGH3* genes suggest that *AtGH3.13* ~ *AtGH3.16* cluster in Arabidopsis was generated by tandem duplication after *Brassica* lineage-specific whole genome triplication and/or other *BoGH3* genes around *BoGH3.13–1*, *BoGH3.13–2*, and *BoGH3.13–4* might have been lost after divergence of *Arabidopsis* and *Brassica* lineages [47]. Consistent with this idea, one intact and one truncated form of *GH3* genes in subgroup 6, *BoGH3.13–1* and *B02g011230*, were identified within 15 kb region on the chromosome 2 of *B. oleracea* var. *oleracea* (Fig. 2b). Members in gene family in plants are known to evolve through both tandem (local) duplication and whole genome duplication, which were followed by gene loss or gene retention leading to functional diversification [48]. Nonetheless, close genomic locations of subgroup 4 and subgroup 6 *GH3* genes in Arabidopsis and *B. oleracea* var. *oleracea* indicate both *AtGH3.12-like* and *AtGh3.13-like GH3* genes were present in proximity before the separation of *Arabidopsis* and *Brassica* lineages. For exon/intron structures of *BoGH3* and *AtGH3* genes, overall similarities were observed for the evolutionarily related genes. However, distributions of RNA-seq reads in NCBI database revealed that protein-coding exons of *BoGH3.1*, *BoGH3.11–2*, and *BoGH3.11–3* are differently organized compared to those of related Arabidopsis *GH3* genes (Fig. 1b). Differences in the structures observed for five *BoGH3* genes (*BoGH3.8–2*, *BoGH3.8–5*, *BoGH3.13–3*, *BoGH3.18–1*, and *BoGH3.18–7*) and related Arabidopsis genes might result from deletions/insertions and incorrect annotations, considering that these five *BoGH3* genes are identified only in Ensembl Plants, not supported by RNA-seq data in NCBI database, and encode predicted GH3 proteins with multiple deletions (Fig. 1b & S1).

Four subgroup 6 *BoGH3* genes, which seem to be generated from same ancestor gene(s), show distinct expression patterns. At the organ level, *BoGH3.13–1* is almost exclusively detected in floral buds by qRT-PCR, while the strongest expressions of *BoGH3.13–3* and *BoGH3.13–4* are observed in siliques (Fig. 4 a, c & d). In case of *BoGH3.13–2*, no significant expression preference is found among different organs and constitutively expressed in all parts of flowers (Figs. 4b, 5 c & f). In developing floral buds, *BoGH3.13–1* is strongly expressed in stamen when floral buds are about 2 ~ 6 mm long (Fig. 5). However, investigation of *BoGH3.13–1* promoter activity using GUS reporter revealed that *BoGH3.13–1* is also expressed in abscission zones in siliques and leaf primordia, in addition to tapetal cells in stamen and pollen grains (Fig. 6f–l). Relatively weak detection of *BoGH3.13–1* in siliques by qRT-PCR may be related to the facts that the gene is expressed only in a small portion of siliques cells, although we do not exclude the possibility that the expression level is also lower in siliques than in stamen. In 2 ~ 6 mm floral buds, in which *BoGH3.13–1* is most strongly expressed, microspores, polarized microspores, and bicellular pollens are mainly observed in anthers (Fig. 7). Similar to *BoGH3.13–1*, two syntenic subgroup 6 Arabidopsis *GH3* genes, *AtGH3.13* and *AtGH3.16,* are expressed in flower stage 9 ~ 11 floral buds and flower stage 12, respectively [49]. More specifically, *AtGH3.16* is expressed in polarized microspore and *AtGH3.13* is expressed bicellular pollens. Based on the numbers of pollen nuclei and floral bud phenotypes [50], the flower stages, when *BoGH3.13–1* is strongly expressed, roughly correspond to stages 8 ~ 12 of Arabidopsis flower and overlap with the periods when *AtGH3.13* and *AtGH3.16* are expressed (Fig. 7 & S2). Although *BoGH3.5–1*, a group II *BoGH3* gene, is also specifically expressed in stamen like *BoGH3.13–1* (Figs. 3a-b & 6u-x), *BoGH3.5–1* seems to be expressed in a longer time period compared to *BoGH3.13–1* (Figs. 5a - b, 6h & o). Different from *BoGH3.13–1*, neither in floral abscission zones nor in leaf primordia is expression of *BoGH3.5–1* observed (Fig. 6 p & r). It needs to be determined which substrate(s) are preferentially used by *BoGH3.13–1* and *BoGH3.5–1*.

*BoGH3.13–1* is not induced by auxin (IAA or 2,4-D), JA, or GA, but expressed in a tissue-specific manner. Different from many *GH3* genes in other plants, which have been found to be induced by various plant hormones [1, 17, 33, 51, 52], no expression changes for *BoGH3.13–1* and 3 other subgroup 6 *BoGH3* genes were detected in our experimental conditions (Fig. 3). In contrast, expression levels of *BoGH3.2* was found to be elevated upon exposure to auxin in the same condition. Similar to our findings, all subgroup 6 *GH3* genes in *B. napus*, an allotetraploid carrying chromosomes with *B. oleracea* origin, did not show any significant expression changes in response to IAA treatment in leaves [33]. Although *BoGH3.13–1* expression is not induced by auxin in our experimental condition, tissues or cells, in which *BoGH3.13–1* promoter activity is detected, largely overlap with the regions where auxin-responsive *DR5* promoter is activated in Arabidopsis and rice (Figs. 3 & 6) [53–56]. We do not exclude the possibility that *BoGH3.13–1* promoter is less sensitive to auxin treatment than *BoGH3.2*, but we prefer the idea that expression of *BoGH3.13–1* is induced by a transcription factor that is activated in tissue-specific manners downstream of auxin signaling pathway. When expression patterns of *BoGH3* genes were probed at the organ level using EMBL-EBI expression atlas (https://www.ebi.ac.uk/gxa/experiments/E-GEOD-42891/Results), *BoGH3.13–1* was found to be specifically expressed in floral buds, similar to our qRT-PCR results (Fig. 4 & Table S3). However, expression levels of *BoGH3.5–1* was found to be higher in silique than in floral bud, different from our results. Although differences in growth conditions and sampling times might have affected gene expressions, transcription profiling based on RNA-seq could have been confounded by sequence reads produced from highly homologous *BoGH3* gene family members. Given that expressions of *BoGH3.13–1* in leaf primordia and floral abscission zone could not be detected by transcription profiling, complete understanding of some *BoGH3* expression patterns seem to require both qRT-PCR and investigation of promoter activity using promoter-reporter system.

Anther-specific expression of *BoGH3.13–1* is directed by 62 bp DNA sequence, from − 340 to − 279 bp from the start codon. Determination of promoter regions important for tissue-specific expressions revealed that about 180 bp P3 region (− 340 ~ − 155) close to the transcription start site is sufficient for anther-specific expression (Fig. 8). The observation that P4 region (− 278 ~ − 155) does not supports anther-specific expression suggests that *cis*-acting element necessary for anther-specific expression is included by 62 bp DNA sequence from − 340 to − 279 bp. GUS expression detected in 5 out of 12 P5 transgenic lines containing − 418 to − 279 bp region further supported this idea. We suspect that deletion of promoter sequences (− 278 ~ − 155) close to the transcription start site makes anther-specific expression depend on the genomic positions where transgene is inserted. In Arabidopsis, Male Sterility 1, a plant homeodomain-finger, and MYB99 transcription factors functioning in anther and pollen development pathway are expressed in microspores, polarized microspores, and bicellular pollens [49, 57]. The findings (1) that *BoGH3.13–1* is strongly expressed when microspores, polarized microspores, and bicellular pollens are produced and (2) that MYB core *cis*-acting element (CTGTTA) is located at − 293 ~ − 288 raises a possibility that *Brassica oleracea var. oleracea* ortholog of Arabidopsis MYB99 plays an important role for anther-specific expression of *BoGH3.13–1* [58]. Because *GUS* expressions in leaf primordia and floral abscission zones are lost without any obvious effect on anther-specific expression, *cis*-acting element important for leaf primordia and floral abscission zone expressions must be located in the − 1489 to − 1017 region in *BoGH3.13–1* promoter and independent of *cis*-acting element for anther-specific expression (Fig. 8a – f).

## Conclusions

In this study, we identified 34 *GH3* genes in *Brassica oleracea var. oleracea*, including four subgroup 6 *GH3* genes, and a critical promoter region for anther-specific expression of a subgroup 6 *BoGH3* gene, *BoGH3.13–1*. The information will broaden our understanding of transcriptional regulations during anther development and can be used to develop transgenic male sterile lines for economically important *Brassica* plants.

## Methods

### Plant growth

*Brassica oleracea var. oleracea* (TO1000 seeds, stock number CS29002) were obtained from the Arabidopsis Biological Resource Center. *Brassica oleracea var. oleracea* and Arabidopsis plants were grown on soil or a half-strength liquid Murashige and Skoog (MS) media (pH 5.7) with vitamins made with Duchefa Biochemie M0222 (Haarlem, Netherlands). Plants were grown under a 16 h (hr) light/8 h dark photoperiod at 22 C°. Organ samples of *Brassica oleracea var. oleracea* were collected from 50-day old soil-grown plants.

Transgenic Arabidopsis plants (ecotype Columbia) carrying *β-glucuronidase* (*GUS*)-coding sequences expressed by *GH3* promoter sequences were selected on half-strength solid MS media containing 0.8% Duchefa Plant agar P1001 (Haarlem, Netherlands) and 20 μg/ml Kanamycin, and transferred to soil for flowering.

### Identification of genes encoding putative GH3 family proteins in *Brassica oleracea var. oleracea*

To identify putative GH3-coding genes in *Brassica oleracea var. oleracea*, 19 *Arabidopsis* GH3 protein sequences downloaded from The Arabidopsis Information Resource (TAIR, http://www.arabidopsis.org/) were used for BLAST search in the Ensembl Plants database (http://plants.ensembl.org/index.html) and the National Centre for Biotechnology Information (NCBI, http://ncbi.nlm.nih.gov). In the Ensembl Plants and NCBI database search, E-value thresholds for candidates were set on 1e^− 1^ and 0.1, respectively. BoGH3 proteins were further determined by the presence of the intact GH3 domains, and their exon/intron structures were determined based on RNA-seq exon coverage and RNA-seq intron spanning reads from NCBI *B. oleracea* annotation Release 100. Similarly, GH3 protein sequences in *B. oleracea* var. *capitata* were identified using the sequence in Bolbase (http://ocri-genomics.org/bolbase/blast/blast.html) [59].

### Multiple sequence alignment and construction of phylogenetic tree

The multiple sequence alignment of GH3 proteins was performed using Clustal Omega and visualized using Jalview [60, 61]. Phylogenetic analysis was performed using the molecular evolutionary genetics analysis (MEGA) software [62]. The evolutionary history was inferred by using maximum likelihood method based on the JTT matrix-based model [63]. All positions with less than 90% site coverage were eliminated. There were a total of 549 positions in the final dataset. The bootstrap test was repeated 1000 times. An orthologous relationship for synteny between *B. oleracea* var. *oleracea* and Arabidopsis was determined using gene information in the Ensembl database (https://plants.ensembl.org/Brassica_oleracea/Info/Index).

### Hormone treatment

For hormone treatment, surface sterilized *Brassica oleracea var. oleracea* seeds were germinated and grown in 24 well plates containing 1 ml half-strength liquid MS media for 5 days. 2,4-D (D0901), IAA (I0901), GA (G0907) and JA (J0936) from Duchefa (Haarlem, Netherlands) were treated to whole seedlings, after the seedlings were further grown in 2 ml fresh liquid media for 6 h.

### Sample collection

Five-day-old seedlings were used to determine whether the *BoGH3* gene of interest is induced by hormone treatment. For gene expression analysis by qRT-PCR, root, leaf, stem, floral bud, open flower, and silique were obtained from 3 individual plants: more specifically, 11th to 13th leaves, fifth to seventh node for stems, a mix of unopened floral buds without white petals exposed (bud length less than about 8 mm), a mix of open flowers (bud length larger than 8 mm) with white petals exposed, and siliques with various sizes were collected. Samples for floral buds were further divided into 5 categories by lengths: 0 ~ 2, 2 ~ 4, 4 ~ 6, 6 ~ 8, and 8 ~ 10 mm sizes (Figure S3). Sepals, petals, anthers, and pistils were collected from 4 ~ 6 mm -long unopened floral buds. After collection, samples were frozen in liquid nitrogen and stored at − 80 C° until RNA isolation. Samples for GUS staining were collected when transgenic Arabidopsis seedlings were 8 days old, or later when inflorescence and siliques were mature enough.

### RNA isolation, reverse transcription, and qRT-PCR analysis

Total RNA was extracted using PhileKorea E-Zol RNA Reagent (Seoul, Korea) or Ambion TRIzol® Reagent (Austin, USA) following the manufacturer’s instructions. For silique samples, Invitrogen Plant RNA Purification Reagent (Carlsbad, USA) was used to. cDNA was synthesized from RNA with 260/280 ratios between 1.8 and 2.1. First stand cDNA was synthesized with Toyobo ReverTra Ace -α (Osaka, Japan) and 1.0 μg of total RNA, according to the manufacturer’s instructions. In case of hormone-treated seedlings, 0.5 μg of total RNA was used. As described in Nam et al. (2019), qRT-PCR was performed with a two-step reaction: 3 min (min) at 95 °C, followed by 50 cycles of 10 s at 95 °C and 30 s at 60 °C. Primer sequences used are listed in Table S4. For each analysis, three technical replicates of at least two independent biological replicates were used.

### Construction of *GH3* promoter-GUS reporter vector and plant transformation

DNA regions upstream of the start codon of *GH3* genes used for promoter analyses are as follows: − 1489 ~ − 1, − 1017 ~ − 1, − 500 ~ − 1, − 418 ~ − 1, − 340 ~ − 155, − 278 ~ − 155 bp of *BoGH3.13–1*, and − 1496 ~ − 1 bp of *BoGH3.5–1*. Putative promoter regions were PCR-amplified with specific primers with SalI or BamHI recognition sequence for cloning (Table S5). After SalI and BamHI digestion, the PCR fragments were cloned into pBI101.1 vector between SalI and BamHI sites. The construct was transformed into Arabidopsis by the floral dip method [64].

### Histochemical GUS staining and paraffin section of GUS-stained samples

Histochemical GUS staining was performed with 0.5 mg/ml MBcell 5-bromo-4-chloro-3-indolyl-beta-D-glucuronic acid-cyclohexylammonium salt (Seoul, Republic of Korea), as previously described [65]. The floral buds of T1 or T2 transgenic plants carrying a *GH3 promoter::GUS* fusion transgene were immersed in GUS reaction buffer in the dark condition for 1 day at 37 °C, after which samples were washed in 95% ethanol for 1 ~ 2 h. At least 7 individual transgenic lines were used to analyze GUS expression patterns.

To perform the paraffin section, GUS-stained samples were fixed in FAA solution (Formaline: ethanol: glacial acetic acid: water = 10: 50: 5: 35) for at least 24 h and washed in water for 24 ~ 48 h. Then the samples were dehydrated in 50, 60, 70, 80, 90% ethanol series for 20 min once, and 100% ethanol for 20 min twice. The samples were incubated in a series of ethanol:xylene mix (75:25, 50:50, and 25:75) for 30 min in each mix, and to a series of xylene:paraffin mix (2:1, 1:1, and 1:2) for 1 h twice in each mix. The samples were incubated in molten paraffin for 24 h and poured into blocks on a slide warmer at 70 °C and cooled down to 25 °C. Eight μm-thick transverse sections of paraffin-embedded samples were made with a microtome. Ribbons of serial sections floated on warm water (50 °C) were transferred to slide glasses on the slide warmer at 70 °C and cooled down to 25 °C. Paraffin in the sections was removed with xylene.

### DAPI staining of pollen grains

For DAPI staining, pollen in 0 ~ 2 mm, 2 ~ 4 mm, 4 ~ 6 mm, 6 ~ 8 mm, and 8 ~ 10 mm TO1000 floral buds were put on microscope slides and stained with several drops of DAPI-staining solution, as described [66]. The pollen nuclei were inspected under an Olympus BX51 fluorescence microscope (Tokyo, Japan) with a DAPI filter.

## Supplementary Information

**Additional file 1: Supplementary Table 1** Protein identifiers and genomic locations of kale-like type *B. oleracea* var. *oleracea* GH3 proteins identified in Ensembl Plants and NCBI database.

**Additional file 2: Supplementary Table 2** GH3 proteins in *B. oleracea* var. *oleracea* and putative orthologs in *B. oleracea* var. *capitata*.

**Additional file 3: Supplementary Table 3** Transcription profiling calculated from high throughput sequencing results.

**Additional file 4: Supplementary Table 4** Sequences of qRT-PCR primers.

**Additional file 5: Supplementary Table 5** Sequences of primers used to clone putative promoter regions of *BoGH3.13–1*.

**Additional file 6: Supplementary Figure 1.** Multiple sequence alignment of thirty-four *B. oleracea* var. *oleracea* and nineteen Arabidopsis GH3 proteins.

**Additional file 7: Supplementary Figure 2.** qRT-PCR results showing expression patterns of three subgroup 4 *BoGH3* genes. qRT-PCR results showing expression patterns in different organs. Relative steady-state expression levels of *BoGH3* genes were determined by qRT-PCR experiment with *Actin* control. Bar graphs show average relative expression values with SEs. The expression level of leaf was set to value 1 and used as reference to compare expression levels in different organs.

**Additional file 8: Supplementary Figure 3.** Morphology of *B. oleracea* var. *oleracea* floral buds used in this study. Upper panels show representative intact floral buds. Lower panels show representative anthers and pistils after sepals and petals were removed. Scale bar shown with fully opened flower is 1 cm.

## Data Availability

The accession numbers of *BoGH3* genes, which were retrieved from Ensembl Plants repository (http://plants.ensembl.org/index.html) and analyzed during the current study, are indicated in parenthesis after gene names: *BoGH3.2* (*Bo1g004760*), *BoGH3.3* (*Bo8g100590*), *BoGH3.5–1* (*Bo1g048130*), *BoGH3.5–2* (*Bo7g111320*), *BoGH3.6–1* (*Bo2g041710*), *BoGH3.6–2* (*Bo3g022080*), *BoGH3.8–2* (*Bo3g023700*), *BoGH3.8–3* (*Bo1g008000*), *BoGH3.8–5* (*Bo7g116230*), *BoGH3.10* (*Bo9g007560*), *BoGH3.11–1* (*Bo4g009300*), *BoGH3.11–2* (*Bo3g039200*), *BoGH3.12–1* (*Bo2g011190*), *BoGH3.12–3* (*Bo9g167830*), *BoGH3.13–1* (*Bo2g011210*), *BoGH3.13–2* (*Bo3g009140*), *BoGH3.13–3* (*Bo7g011450*), *BoGH3.13–4* (*Bo9g166800*), *BoGH3.17–3* (*Bo8g039460*), *BoGH3.18–1* (*Bo4g164910*), *BoGH3.18–2* (*Bo9g052150*), *BoGH3.18–3* (*Bo9g117680*), *BoGH3.18–5* (*Bo8g109440*), *BoGH3.18–6* (*Bo8g109480*), and *BoGH3.18–7* (*Bo8g109490*). The accession numbers of BoGH3 proteins, which were retrieved from NCBI repository (https://www.ncbi.nlm.nih.gov/) and analyzed during the current study, are indicated in parenthesis after protein names: BoGH3.1 (XP_013608568.1), BoGH3.8–1 (XP_013619802.1), BoGH3.8–4 (XP_013596331.1), BoGH3.9 (XP_013632208.1), BoGH3.11–3 (XP_013632135.1), BoGH3.12–2 (XP_013623633.1), BoGH3.17–1 (XP_013583597.1), BoGH3.17–2 (XP_013594064.1), and BoGH3.18–4 (XP_013603489.1). Accession numbers of *BoGH3* proteins with additional information are also found in Table S1. RNA-seq data for transcription profiling shown in Table S3 was retrieved from GEO repository (https://www.ncbi.nlm.nih.gov/geo/query/acc.cgi?acc=GSE42891).

## Abbreviations

*GH3*: *Gretchen Hagen 3*
bp: Base pairs
qRT-PCR: Quantitative reverse transcription polymerase chain reaction
GUS: β-glucuronidase
DAPI: 4′,6-diamidino-2-phenylindole
2,4-D: 2,4-Dichlorophenoxy acetic acid
IAA: Indole-3-acetic acid
GA: Gibberellic acid 3
JA: Jasmonic acid
