# Supplementary material for: Genome-wide identification of GH3 genes in Brassica oleracea and identification of a promoter region for anther-specific expression of a GH3 gene
Source: BMC Genomics. 2021 Jan 6;22:22. doi: 10.1186/s12864-020-07345-9 (PMC7789250; doi:10.1186/s12864-020-07345-9)
Supplement: Supplementary file 1 — Additional file 1: Supplementary Table 1 Protein identifiers and genomic locations of kale-like type B. oleracea var. oleracea GH3 proteins identified in Ensembl Plants and NCBI database. [file 12864_2020_7345_MOESM1_ESM.docx]

**Table S1.** GH3 proteins in TO1000, *B. oleracea* var. *oleracea.*

| **GH3 proteins in this study (Gene identifier, length in aa)** | **Protein name in Ensembl Plants**  **(length in aa)** | **Chromosomal location in of gene in Ensembl plants** | **Protein name in NCBI (length in aa)** | **Chromosomal location of gene in NCBI (Number of exons)** |
| --- | --- | --- | --- | --- |
| BoGH3.1  (Bo9g023750,590)^1^ | Bo9g023750 (632) | C9:7288877..7290990 | XP_013608568.1 (590) | C9:7288646..7290985 |
| BoGH3.2  (Bo1g004760  ,587) | Bo1g004760 (587) | C1:963781..965912 | XP_013619655.1 (587) | C1:963628..965982 |
| BoGH3.3  (Bo8g100590,596) | Bo8g100590 (596) | C8:34980097..34982319 | XP_013603160.1 (596) | C8:34980059..34982419 |
| BoGH3.5-1  (Bo1g048130,612) | Bo1g048130 (612) | C1:13770561..13773075 | XP_013620677.1 (612) | C1:13770505..13773269 |
| BoGH3.5-2  (Bo7g111320, 612) | Bo7g111320 (612) | C7:44255309..44257649 | XP_013593295.1 (612) | C7:44255157..44257854 |
| BoGH3.6-1  (Bo2g041710,612) | Bo2g041710 (612) | C2:11252172..11254389 | XP_013616370.1 (612) | C2:11252080..11254558 |
| BoGH3.6-2  (Bo3g022080,612) | Bo3g022080 (612) | C3:7549186..7551295 | XP_013623669.1 (612) | C3:7549096..7551427 |
| BoGH3.8-1  (Bo2g050470,577)^1^ | Bo2g050470 (547) | C2:14055016..14057037 | XP_013619802.1 (577) | C2:14055016..14057274 |
| BoGH3.8-2  (Bo3g023700,548)^2^ | Bo3g023700 (548) | C3:8633021..8635250 | N.I. | N.I. |
| BoGH3.8-3  (Bo1g008000,581) | Bo1g008000 (581) | C1:2786075..2788529 | XP_013589028.1 (581) | C1:2786067..2788625 |
| BoGH3.8-4  (Bo7g116220,572)^1^ | Bo7g116220 (513) | C7:46376042..46378757 | XP_013596331.1 (572) | C7:46375974..46378779 |
| BoGH3.8-5  (Bo7g116230,485)^2^ | Bo7g116230 (485) | C7:46381204..46383374 | N.I. | N.I. |
| BoGH3.9  (Bo4g002340,594)^1^ | Bo4g002340 (615) | C4:188039..190721 | XP_013632208.1 (594) | C4:187528..190873 |
| BoGH3.10  (Bo9g007560,591) | Bo9g007560 (591) | C9:1571618..1574312 | XP_013612597.1 (591) | C9:1571583..1574490 |
| BoGH3.11-1  (Bo4g009300,579) | Bo4g009300 (579) | C4:1068973..1071578 | XP_013636858.1 (579), | C4:1068788..1072423 |
| BoGH3.11-2  (Bo3g039200,578) | Bo3g039200 (578) | C3:15628677..15630963 | XP_013621759.1 (578) | C3:15627738..15631135 |
| BoGH3.11-3  (Bo4g196110,576)^1^ | Bo4g196110 (619) | C4:53178320..53180921 | XP_013632135.1 (576) | C4:53178284..53180956 |
| BoGH3.12-1  (Bo2g011190, 570) | Bo2g011190 (570) | C2:2966297..2968472 | XP_013620838.1 (570) | C2:2966267..2968532 |
| BoGH3.12-2  (Bo3g009110,573)^1^ | Bo3g009110 (589) | C3:3055286..3060566 | XP_013623633.1 (573) | C3:3055074..3060718 |
| BoGH3.12-3  (Bo9g167830,577) | Bo9g167830 (577) | C9:49535453..49537674 | XP_013606484.1 (577) | C9:49535338..49539591 |
| BoGH3.13-1  (Bo2g011210,595) | Bo2g011210 (595) | C2:2976627..2979715 | XP_013620205.1 (595) | C2:2976608..2979958 |
| BoGH3.13-2  (Bo3g009140,593) | Bo3g009140 (593) | C3:3074165..3076899 | XP_013628487.1 (593) | C3:3073982..3077077 |
| BoGH3.13-3  (Bo7g011450,570)^2^ | Bo7g011450 (570) | C7..3830741..3833511 | N.I. | N.I. |
| BoGH3.13-4  (Bo9g166800,594) | Bo9g166800 (594) | C9:49515722..49518206 | XP_013610157.1 (594) | C9:49515687..49518297 |
| BoGH3.17-1  (Bo5g053450,609)^1^ | Bo5g053450 (659) | C5:17593754..17596403 | XP_013583597.1 (609) | C5:17593745..17596529 |
| BoGH3.17-2  (Bo7g048440,608)^1^ | Bo7g048440 (631) | C7:17881583..17884578 | XP_013594064.1 (608) | C7:17881537..17884698 |
| BoGH3.17-3  (Bo8g039460,604) | Bo8g039460 (604) | C8:13694402..13698434 | XP_013603490.1 (604) | C8:13694262..13698482 |
| BoGH3.18-1  (Bo4g164910,484)^2^ | Bo4g164910 (484) | C4:44119367..44122217 | N.I. | N.I. |
| BoGH3.18-2  (Bo9g052150,561) | Bo9g052150 (561) | C9:15733799..15736766 | XP_013609445.1 (561), | C9:15733710..15736908 |
| BoGH3.18-3  (Bo9g117680,576) | Bo9g117680 (576) | C9:37416261..37418409 | XP_013610407.1 (561) | C9:37416082..37418364 |
| BoGH3.18-4  (Bo8g109430,585)^1^ | Bo8g109430 (571) | C8:39212890..39214972 | XP_013603489.1 (585) | C8:39212848..39214972 |
| BoGH3.18-5  (Bo8g109440,572)^2^ | Bo8g109440 (572) | C8:39222877..39224989 | N.I. | N.I. |
| BoGH3.18-6  (Bo8g109480,567) | Bo8g109480 (567) | C8:39241406..39243693 | XP_013603699.1 (567) | C8:39241406..39243693 |
| BoGH3.18-7  (Bo8g109490,475)^2^ | Bo8g109490 (475) | C8:39245140..39247123 | N.I. | N.I. |

N.I. : Not identified. ^1^: Coding sequences were different between NCBI and Ensembl Plants (<http://plants.ensembl.org/index.html>) and the gene models in NCBI database (<http://ncbi.nlm.nih.gov>) were adopted. ^2^: Corresponding gene models were not found in in NCBI database.
