# Supplementary material for: Genome-wide identification of GH3 genes in Brassica oleracea and identification of a promoter region for anther-specific expression of a GH3 gene
Source: BMC Genomics. 2021 Jan 6;22:22. doi: 10.1186/s12864-020-07345-9 (PMC7789250; doi:10.1186/s12864-020-07345-9)
Supplement: Supplementary file 2 — Additional file 2: Supplementary Table 2 GH3 proteins in B. oleracea var. oleracea and putative orthologs in B. oleracea var. capitata. [file 12864_2020_7345_MOESM2_ESM.docx]

**Table S2.** Putative GH3 proteins in *B. oleracea* var. *oleracea* and putative orthologs in *B. oleracea* var. *capitata.*

| Name in *B. oleracea* var. *oleracea*) | Length in aa | Chromosomal location of gene in Ensembl plants | Names of putative orthologs in  *B. oleracea* var. *capitata*^1^ | Length in aa | Chromosomal location of gene^1^ |
| --- | --- | --- | --- | --- | --- |
| BoGH3.2 | 587 | C1:963781..965912 | Bol028920 | 586 | C01:855204..857325 |
| BoGH3.8-3 | 581 | C1:2786075..2788529 | Bol013595 | 657 | C01:2681854..2685346 |
| BoGH3.5-1 | 612 | C1:13770561..13773075 | Bol013052 | 612 | C01:13414608..13417122 |
| BoGH3.12-1 | 570 | C2:2966297..2968472 | N.I. |  |  |
| BoGH3.13-1 | 595 | C2:2976627..2979715 | Bol004283 | 577 | Scaffold000328:411919..414994 |
| BoGH3.6-1 | 612 | C2:11252172..11254389 | Bol018258 | 612 | C02:11104094..11106307 |
| BoGH3.8-1 | 577 | C2:14055016..14057037 | N.I. |  |  |
| BoGH3.12-2 | 573 | C3:3055286..3060566 | Bol034228 | 573 | C03:2200126..2202141 |
| BoGH3.13-2 | 593 | C3:3074165..3076899 | Bol034231 | 593 | C03:2231917..2234643 |
| BoGH3.6-2 | 612 | C3:7549186..7551295 | Bol027952 | 612 | C03:6694176..6696277 |
| BoGH3.9-2^2^ | 548 | C3:8633021..8635250 | N.I. |  |  |
| BoGH3.11-2 | 578 | C3:15628677..15630963 | Bol029586 | 578 | C03:13631557..13633943 |
| BoGH3.9 | 594 | C4:188039..190721 | N.I. |  |  |
| BoGH3.11-1 | 579 | C4:1068973..1071578 | Bol000924 | 579 | Scaffold000489:59364:61982 |
| BoGH3.18-1^2^ | 484 | C4:44119367-44122217 | N.I. |  |  |
| BoGH3.11-3 | 576 | C4:53178320..53180921 | Bol021779 | 576 | C04:40607452..40609429 |
| BoGH3.17-1 | 609 | C5:17593754..17596403 | N.I. |  |  |
| BoGH3.13-3^2^ | 570 | C7:3830741..3833511 | N.I. |  |  |
| BoGH3.17-2 | 608 | C7:17881583..17884578 | Bol041811 | 608 | C07:16555730..16558611 |
| BoGH3.5-2 | 612 | C7:44255309..44257649 | Bol042327 | 612 | C07:44390687..44393005 |
| BoGH3.8-4 | 572 | C7:46376042..46378757 | Bol033825 | 572 | C07:46608869..46611158 |
| BoGH3.8-5^2^ | 485 | C7:46381204-46383374 | N.I. |  |  |
| BoGH3.17-3 | 604 | C8:13694402..13698434 | Bol028316 | 604 | C08:12742767..12746797 |
| BoGH3.3 | 596 | C8:34980097..34982319 | N.I. |  |  |
| BoGH3.18-4 | 585 | C8:39212890..39214972 | N.I. |  |  |
| BoGH3.18-5^2^ | 572 | C8:39222877..39224989 | N.I. |  |  |
| BoGH3.18-6 | 567 | C8:39241406..39243693 | N.I. |  |  |
| BoGH3.18-7^2^ | 475 | C8:39245140-39247123 | N.I. |  |  |
| BoGH3.10 | 591 | C9:1571618..1574312 | Bol032157 | 591 | C09:1594850..1597548 |
| BoGH3.1 | 590 | C9:7288877..7290990 | Bol019349 | 590 | C09:6041789..6043776 |
| BoGH3.18-2 | 561 | C9:15733799..15736766 | N.I. |  |  |
| BoGH3.18-3 | 576 | C9:37416261..37418409 | N.I. |  |  |
| BoGH3.13-4 | 594 | C9:49515722..49518206 | Bol043438 | 594 | C09:35488766..35491249 |
| BoGH3.12-3 | 577 | C9:49535453..49537674 | Bol043441 | 577 | C09:35516908..35519132 |
|  |  |  | Bol028089^3^ | 470 | C03:7849806..7851771 |
|  |  |  |  |  |  |
|  |  |  | Bol016724^3^ | 580 | C06:26469501..26474856 |
|  |  |  | Bol041490^3^ | 544 | C07:10708719..10711504 |
|  |  |  | Bol045809^3^ | 592 | C08:34864965..34867194 |
|  |  |  | Bol031266^3^ | 585 | C08:39282672..39284801 |
|  |  |  | Bol031263^3^ | 571 | C08:39312834..39314843 |
|  |  |  | Bol038891^3^ | 510 | C09:23060907..23065342 |
|  |  |  | Bol004286^3^ | 536 | Scaffold000328:443018..445199 |
|  |  |  | Bol001121^3^ | 615 | Scaffold000467:56453:59137 |

N.I. : Not identified based on BLAST search in Bolbase (<http://ocri-genomics.org/bolbase/blast/blast.html>) with protein sequences and cutoff of 95% identities. ^1^: Gene name and chromosomal location at Bolbase (Yu *et al*., 2013). ^2^: Gene model in NCBI was either absent or incorrect. ^3^: These GH3 genes were identified only in Bolbase.
