# Supplementary material for: Genome-wide identification of GH3 genes in Brassica oleracea and identification of a promoter region for anther-specific expression of a GH3 gene
Source: BMC Genomics. 2021 Jan 6;22:22. doi: 10.1186/s12864-020-07345-9 (PMC7789250; doi:10.1186/s12864-020-07345-9)
Supplement: Supplementary file 3 — Additional file 3: Supplementary Table 3 Transcription profiling calculated from high throughput sequencing results. [file 12864_2020_7345_MOESM3_ESM.docx]

**Table S3**. Transcription profiling calculated from high throughput sequencing results.

Data was extracted from EMBL-EBI expression atlas (<https://www.ebi.ac.uk/gxa/experiments/E-GEOD-42891/Results>). RNA-seq experiments for raw data in the expression atlas is described in reference 47.

| **Gene name** | **Root** | **Leaf** | **Floral bud** | **Open flower** | **Silique** | **Callus** | |
| --- | --- | --- | --- | --- | --- | --- | --- |
| *BoGH3.1* | 1 | 1 | 10 | 4 |  | 1 |  |
| *BoGH3.2* | 41 | 1 | 2 | 0.7 | 8 | 13 |  |
| *BoGH3.3* |  | 1 | 3 |  | 4 |  |  |
| *BoGH3.5-1* | 3 |  | 7 |  | 17 | 48 |  |
| *BoGH3.5-2* | 5 |  | 5 | 2 | 7 | 62 |  |
| *BoGH3.6-1* | 4 | 3 | 9 | 26 | 0.7 | 12 |  |
| *BoGH3.6-2* | 0.5 | 1 | 14 | 3 | 1 |  |  |
| *BoGH3.8-1* |  |  |  |  |  |  |  |
| *BoGH3.8-2* |  |  |  |  |  |  |  |
| *BoGH3.8-3* |  |  |  |  |  |  |  |
| *BoGH3.8-4* | 8 |  | 2 |  |  | 2 |  |
| *BoGH3.8-5* |  | 17 | 2 | 0.9 | 1 | 4 |  |
| *BoGH3.9* |  |  | 2 |  | 47 | 0.6 |  |
| *BoGH3.10* | 13 | 11 | 29 | 24 | 35 | 17 |  |
| *BoGH3.11-1* | 227 | 6 | 14 | 15 | 38 | 164 |  |
| *BoGH3.11-2* | 120 | 2 | 9 | 30 | 7 | 31 |  |
| *BoGH3.11-3* | 3 |  | 1 | 0.6 | 2 | 3 |  |
| *BoGH3.12-1* | 2 |  |  |  |  | 4 |  |
| *BoGH3.12-2* | 1 |  |  |  | 2 | 2 |  |
| *BoGH3.12-3* | 3 | 0.7 | 0.9 | 0.8 | 10 | 9 |  |
| *BoGH3.13-1* |  |  | 221 |  |  |  |  |
| *BoGH3.13-2* | 37 | 27 | 38 | 10 | 44 | 68 |  |
| *BoGH3.13-3* |  |  |  |  |  |  |  |
| *BoGH3.13-4* |  |  | 4 |  |  |  |  |
| *BoGH3.17-1* |  |  | 2 | 19 |  |  |  |
| *BoGH3.17-2* | 1 |  | 7 | 7 | 0.6 | 9 |  |
| *BoGH3.17-3* |  |  |  |  | 9 |  |  |
| *BoGH3.18-1* |  |  |  |  |  |  |  |
| *BoGH3.18-2* | 0.8 |  |  |  |  | 0.9 |  |
| *BoGH3.18-3* | 1 |  |  |  |  |  |  |
| *BoGH3.18-4* |  |  |  |  |  |  |  |
| *BoGH3.18-5* |  |  |  |  |  |  |  |
| *BoGH3.18-6* |  |  |  |  |  |  |  |
| *BoGH3.18-7* |  |  |  |  |  |  |  |
