# Supplementary material for: Genome-wide identification of GH3 genes in Brassica oleracea and identification of a promoter region for anther-specific expression of a GH3 gene
Source: BMC Genomics. 2021 Jan 6;22:22. doi: 10.1186/s12864-020-07345-9 (PMC7789250; doi:10.1186/s12864-020-07345-9)
Supplement: Supplementary file 4 — Additional file 4: Supplementary Table 4 Sequences of qRT-PCR primers. [file 12864_2020_7345_MOESM4_ESM.docx]

**Supplementary Table 4.** Sequences of qRT-PCR primers.

| **Gene name** | **Forward primer (5’ – 3’)** | **Reverse primer (5’ – 3’)** | **bp** | **Tm (℃)** | **GC content (%)** | **Primer location** |
| --- | --- | --- | --- | --- | --- | --- |
| *Actin* | GTTGCACCAAGCAGCATGAA | AGAATGGAACCACCGATCCA | 81 bp | 63.8 / 63.3 | 50.0 / 50.0 | Exon3 / Exon3 |
| *BoGH3.2* | CGATTGGTCCGTTGGAGATAC | ACCTCGGGACCTTATACTGATTA | 108 bp | 62.2 / 62.2 | 52.4 / 43.5 | Exon3 / Exon3 |
| *BoGH3.5-1* | GCATCTGTCTCTGAGTACACG | AACAGTGTTGAGAGACTCTTCTAG | 156 bp | 61.8 / 61.6 | 52.4 / 41.7 | Exon3 / Exon3 |
| *BoGH3.12-1* | ATGCAGACACCTCAACGT | ACACTTGGAGAATGCCTCTTC | 107 bp | 61.3 / 62.2 | 50.0 / 47.6 | Exon4 / Exon4 |
| *BoGH3.13-1* | TCGTACACCATTGTTCCAAGT | ACAGGGTTCTTTGTGGGATTAT | 95 bp | 61.9 / 62 | 42.9 / 40.9 | Exon4 / Exon4 |
| *BoGH3.12-2* | GTTTAAGGCGTTGAGTCAAGC | GTAGTGACCCGGAAATGTTGA | 103 bp | 61.8 / 62.1 | 47.6 / 47.6 | Exon5 / Exon5 |
| *BoGH3.13-2* | GATCTCCTCAAAGCCGTAACAA | CACGTAGTGTCCTGGAAATGAA | 111 bp | 62.4 / 62.3 | 45.5 / 45.5 | Exon5 / Exon5 |
| *BoGH3.13-3* | TATTGGGAACTCGGGTGTAAAG | TCATTCCCTCGTCCATTTCTG | 125 bp | 61.9 / 61.9 | 45.5 / 47.6 | Exon5 / Exon5 |
| *BoGH3.13-4* | GAGTTCTATGGTGGTGGTCTTC | AAGGCTTGCTCAGAGGATTTAT | 93 bp | 62 / 62 | 50.0 / 40.9 | Exon4 / Exon4 |
| *BoGH3.12-3* | GCCTTGGACCTCAACAAAGA | GTCTCTTCACTCACCACTCTTATC | 137 bp | 62.4 / 61.9 | 50.0 / 45.8 | Exon4 / Exon4 |

Tm: melting temperature. Two numbers for Tm, GC content, and primer locations are those for forward and reverse primer, respectively.

Note that primers for *Actin* genes do not differentiate *Bo1g116200*, *Bo3g071760* and *Bo5g117040* (Aiamla-or *et al*., 2012)
