# Supplementary material for: Genome-wide identification of GH3 genes in Brassica oleracea and identification of a promoter region for anther-specific expression of a GH3 gene
Source: BMC Genomics. 2021 Jan 6;22:22. doi: 10.1186/s12864-020-07345-9 (PMC7789250; doi:10.1186/s12864-020-07345-9)
Supplement: Supplementary file 5 — Additional file 5: Supplementary Table 5 Sequences of primers used to clone putative promoter regions of BoGH3.13–1. [file 12864_2020_7345_MOESM5_ESM.docx]

**Supplementary Table 5**. Sequences of primers used to clone putative promoter regions of *BoGH3.13-1*.

| **Promoter name:**  **DNA coverage** ^a^ | **Forward primer sequence (5’ to 3’)** | **Reverse primer sequence (5’ to 3’)** | **Size** |
| --- | --- | --- | --- |
|  |  |  |  |
| P0: 1489 ~ -1 | AAAGTCGACCTATTTTTGATTCGTAAATGGTGGA | AAGGATCCGGTTTTCTTCTTTTTTGATTATCTGCA | 1489 bp |
| P1: -1017 ~ -1 | AAAGTCGACCACAAAGAGGTCCAATAATTTCT | AAGGATCCGGTTTTCTTCTTTTTTGATTATCTGCA | 1017 bp |
| P2: -500 ~ -1 | AAAGTCGACTGAACCGAATTTGTTTCCG | AAGGATCCGGTTTTCTTCTTTTTTGATTATCTGCA | 500 bp |
| P3: -418 ~ -1 | AAAGTCGACTCAGGGAGAGTTTTCAGGAC | AAGGATCCGGTTTTCTTCTTTTTTGATTATCTGCA | 418 bp |
| P4: -418 ~ -279 | AAAGTCGACTCAGGGAGAGTTTTCAGGAC | AAGGATCCGGTTTTCTTCTTTTTTGATTATCTGCA | 140 bp |
| P5: -340 ~ -155 | AAAGTCGACGTTTATGTTTGTGCTGATAC | AAGGATCCTCCGTAGATGAGTTGTGGCCTCT | 186 bp |
| P6: -278 ~ -155 | AAAGTCGACGAA ACTCATCCAGCGCTTCC | AAGGATCCTCCGTAGATGAGTTGTGGCCTCT | 124 bp |
|  |  |  |  |

^a^: -1 is defined as the first nucleotide in the upstream of the translation start codon

Underlined text represents restriction enzyme recognition sites used for cloning.
