# Supplementary figures and images for: Genome-wide identification of GH3 genes in Brassica oleracea and identification of a promoter region for anther-specific expression of a GH3 gene
Source: BMC Genomics. 2021 Jan 6;22:22. doi: 10.1186/s12864-020-07345-9 (PMC7789250; doi:10.1186/s12864-020-07345-9)

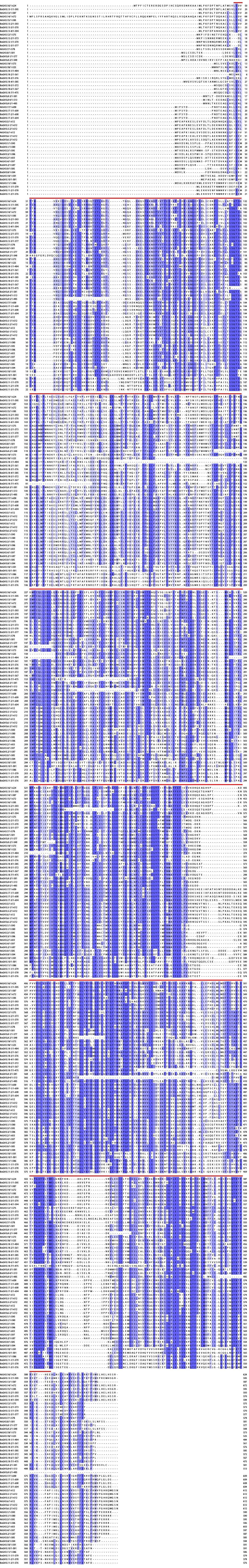

Supplement: Supplementary file 6 — Additional file 6: Supplementary Figure 1. Multiple sequence alignment of thirty-four B. oleracea var. oleracea and nineteen Arabidopsis GH3 proteins. [file 12864_2020_7345_MOESM6_ESM.jpg]

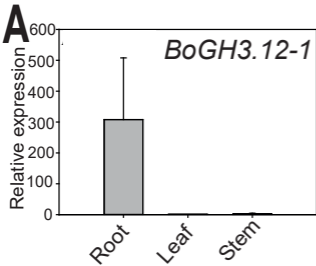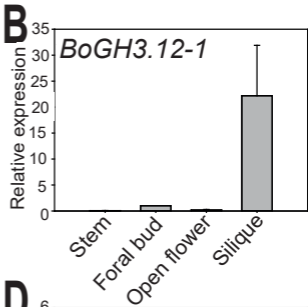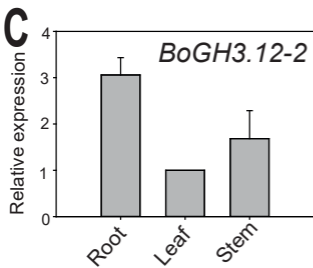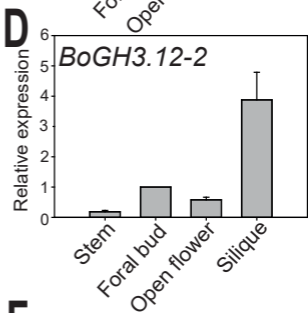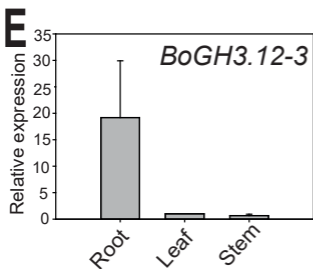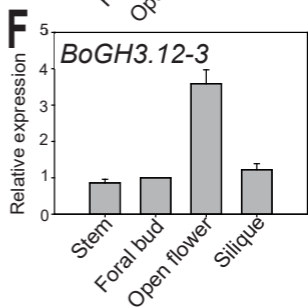

Supplement: Supplementary file 7 — Additional file 7: Supplementary Figure 2. qRT-PCR results showing expression patterns of three subgroup 4 BoGH3 genes. qRT-PCR results showing expression patterns in different organs. Relative steady-state expression levels of BoGH3 genes were determined by qRT-PCR experiment with Actin control. Bar graphs show average relative expression values with SEs. The expression level of leaf was set to value 1 and used as reference to compare expression levels in different organs. [file 12864_2020_7345_MOESM7_ESM.pdf]

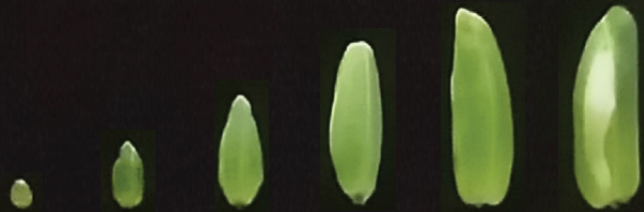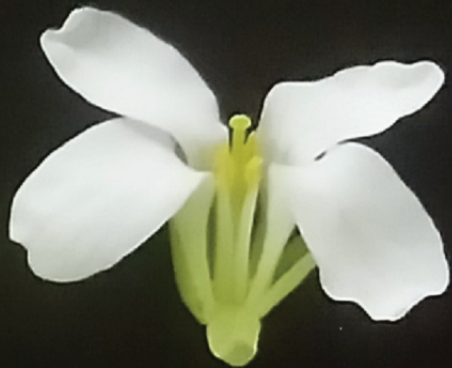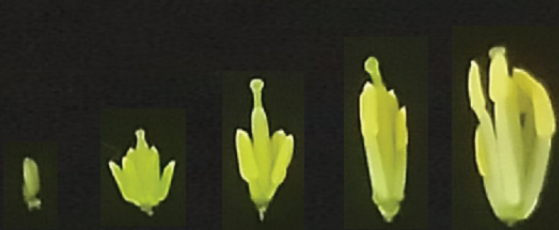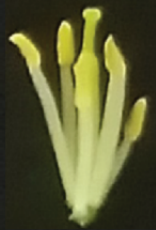

1 mm

3 mm

5 mm

7 mm

9 mm  
(unopened)

9 mm  
(opened)

Opened  
flower

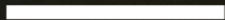

Supplement: Supplementary file 8 — Additional file 8: Supplementary Figure 3. Morphology of B. oleracea var. oleracea floral buds used in this study. Upper panels show representative intact floral buds. Lower panels show representative anthers and pistils after sepals and petals were removed. Scale bar shown with fully opened flower is 1 cm. [file 12864_2020_7345_MOESM8_ESM.pdf]
